# Children with autoimmune hepatitis receiving standard-of-care therapy demonstrate long-term obesity and linear growth delay

**DOI:** 10.1097/HC9.0000000000000624

**Published:** 2025-02-03

**Authors:** Or Steg Saban, Shannon M. Vandriel, Syeda Aiman Fatima, Celine Bourdon, Amrita Mundh, Vicky L. Ng, Simon C. Ling, Robert H.J. Bandsma, Binita M. Kamath

**Affiliations:** 1 Division of Gastroenterology, Hepatology and Nutrition, The Hospital for Sick Children and the University of Toronto, Toronto, Ontario, Canada; 2 Translational Medicine Program, The Hospital for Sick Children, Toronto, Ontario, Canada; 3 Department of Nutritional Sciences, Faculty of Medicine, University of Toronto, Toronto, Ontario, Canada; 4 Division of Gastroenterology, Hepatology and Nutrition, Children’s Hospital of Philadelphia and the University of Pennsylvania Perelman School of Medicine, Philadelphia, Pennsylvania, USA

**Keywords:** corticosteroids, growth, height, metabolic syndrome, overweight

## Abstract

**Background:**

Standard-of-care therapy in children with autoimmune hepatitis (AIH) includes induction with prednisone 1–2 mg/kg daily with gradual weaning of the dose. We aimed to test the hypothesis that children with AIH receiving standard-of-care treatment have altered growth trajectories.

**Methods:**

Children diagnosed with AIH between 1997 and 2023 at SickKids had serial growth measurements. Mixed effect models assessed the impact of time and daily steroid exposure on *z*-scores. Kaplan-Meier survival methods were used to estimate the cumulative incidence of new-onset growth impairments. A time-dependent Cox proportional hazards model was constructed to determine predictors for growth impairments.

**Results:**

Sixty-one children (66% females, median age at diagnosis 11.5 y) were included. BMIz showed a sharp increase, and HAZ declined significantly without returning to baseline. Each 1 mg/kg/d prednisone exposure increased BMIz gain in the first 6 months by 0.27 ([95% CI: 0.11, 0.42], *p* = 0.001), and decreased HAZ by −0.02 ([95% CI: −0.03, −0.01], *p* = 0.005). Children diagnosed before puberty exhibited a higher occurrence of excessive weight gain (72.2% vs. 49.3%; log-rank *p* < 0.01) and obesity (63% vs. 31.5%; log-rank *p* < 0.01) compared to those diagnosed during puberty. In a Cox proportional-hazards model, young age at diagnosis and daily prednisone dose >10 mg 6 months after diagnosis were predictors for linear growth delay.

**Conclusions:**

This study demonstrates that children with AIH receiving standard-of-care therapy demonstrate altered growth trajectories, long-term excess weight gain, obesity, and linear growth delay. Young age at diagnosis and >10 mg of daily prednisone at 6 months are predictors for linear growth delay. These data indicate the need to re-evaluate standard treatment algorithms for pediatric AIH in terms of steroid dosing and potential nonsteroid alternatives.

## Graphical abstract

**Figure:**
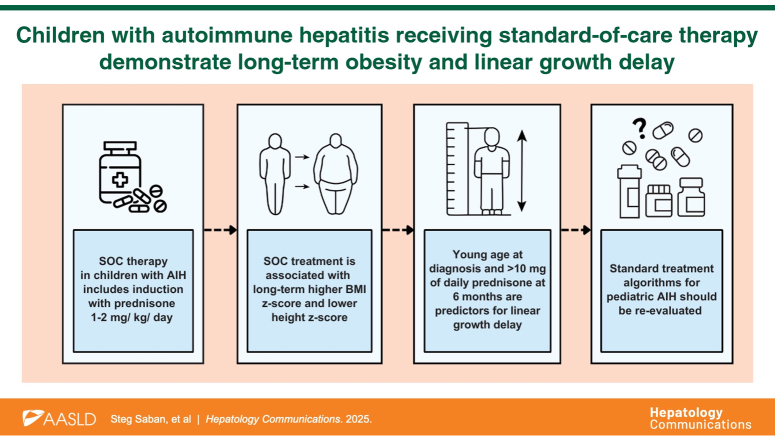

## INTRODUCTION

Autoimmune hepatitis (AIH) is a chronic inflammatory liver disease that presents more severely in children compared to adults. At diagnosis, 38% of pediatric patients have cirrhosis.^1^,^2^ The aggressive course of the disease, coupled with potential delays in diagnosis and treatment, negatively impacts the long-term prognosis, underscoring the rationale for prompt pharmacological intervention in children.^3^ First-line therapies aim to improve symptoms, control hepatic inflammation, achieve biochemical remission, prevent disease progression, and promote fibrosis regression.

The standard-of-care (SOC) induction phase of AIH treatment in children includes prednisone, 1–2 mg/kg daily (maximum dose 40–60 mg daily), in combination with azathioprine which is usually initiated 2–4 weeks after prednisone.^3^ Prednisone is gradually weaned over the next 6 months to a lower dose of 5–10 mg daily that aims to achieve biochemical remission (defined as the normalization of serum ALT, AST, and IgG levels^4^). Thereafter, patients are typically maintained on azathioprine with or without low-dose prednisone (between 0 and 5 mg daily). It is well established that prednisone therapy is associated with adverse effects, including increased weight gain, glucose intolerance, hypertension, fatty liver, osteoporosis, and opportunistic infections.^5^ Weight gain during the induction phase of treatment is the most frequent of these adverse effects and is expected. Families are generally counseled to this effect and told that this excess weight is lost during the maintenance phase of treatment. We hypothesized that some children do not revert to their baseline growth trajectory following standard prednisone exposure during induction and maintenance therapy and that SOC AIH treatment promotes longer-term obesity. While 2 small studies have explored growth in relation to steroid exposure in children treated for AIH, neither addressed growth trajectories with SOC regimens.^6^,^7^ This study aimed to evaluate the long-term impact of SOC AIH therapy on growth and weight gain in children.

## METHODS

This was a retrospective single-center study performed at the Hospital for Sick Children (SickKids) in Toronto, Canada. Participants were ascertained using diagnostic codes for AIH and patients’ records were obtained from the electronic medical charts and screened for eligibility. The study was approved by the research ethics committee at SickKids (REB 1000080493).

### Eligibility criteria

Patients meeting the following criteria were included: (1) patients diagnosed with AIH and followed at SickKids between January 1, 1997, and January 31, 2023, (2) with a confirmatory biopsy for AIH, and (3) documented follow-up for 1 year or more. Patients were excluded if their treatment regimen was associated with extra or nonstandard prednisone exposure, specifically: (1) not treated with SOC treatment (1–2 mg/kg/d, 60 mg daily max), or if they (2) received i.v. corticosteroids for more than 10 days, or (3) had more than 1 flare in a year. In addition, patients were excluded if they (4) had biliary duct injury (evidence of sclerosing cholangitis).

### Study participants

Data retrieved from electronic and paper medical records included: biological sex, prematurity, ethnicity, social worker involvement (as a marker for socioeconomic status), and inflammatory bowel disease (IBD) comorbidity. Age at diagnosis was defined as the day AIH was confirmed with a liver biopsy. Initiation of puberty (Tanner stage 2) was estimated based on age (girls: 10.3 y, boys: 10.8 y),^8^ as these data were not available. The type of AIH was defined as documented in the clinic note. If the type was undefined, patients with anti–liver-kidney microsomal antibody (anti-LKM) 1:40 or greater were labeled as AIH type 2. Anti–nuclear antibody (ANA), anti–smooth muscle antibody (Anti SMA), anti–LKM, and IgG levels were recorded. Data were collected from clinic visits at the time of the diagnosis, every 6 months for 2 years, and then annually for up to 5 years from diagnosis. Weight and height measurements were recorded, and body mass index (BMI) was calculated. Age- and sex-standardized *z*-scores for BMI (BMIz) and height-for-age (HAZ) were derived using WHO growth charts for Canada.^9^ Liver biopsy reports at the time of diagnosis were reviewed, and activity score and fibrosis stage were recorded. In our hospital, the Scheuer Scoring System is routinely used for grading and staging.^10^ Overweight status and obesity were defined as BMI ≥85%, *z*- score >1 and BMI ≥95%, *z*-score >1.64 respectively.^11^,^12^ Excessive weight gain was defined as an increase of 1.0 or more in BMIz at a given visit, relative to the diagnosis time point; while linear growth delay was defined as a decrease of 1.0 or more HAZ at a given visit.^13^,^14^ Daily prednisone dose at 6 months, cumulative steroid dose at 6 months (g), and average daily exposure over 6 months (mg/kg/d) were calculated. The steroid weaning plan was guided by blood work in the hospital and also in local laboratories, to allow quicker weaning. Number of flares per patient was also recorded. Data regarding other medications were also collected, including metformin, amlodipine, GLP-1 agonists, and statins.

### Statistical analysis

Continuous variables are presented as median with IQR and categorical variables as n (%). The Fisher exact test was used to compare categorical variables. Mann-Whitney *U* test was used to determine differences between group means of nonrelated samples. Piecewise regression models were built to assess changes in growth parameters (ie, BMIz and HAZ) in the early period after diagnosis (before 6 mo) versus the later period following diagnosis (after 6 mo). These models included a one-knot point positioned at 6 months after diagnosis for BMI and 12 months after diagnosis for height. Knot positions were chosen based on an assessment of the inflection point, the time of planned visits, and model fit metrics. Differences in slopes before or after the knot point were calculated. Using emmeans R package, marginal means were derived, and differences were tested between the time of diagnosis and 6 and 48 months for BMIz or at 12 and 48 for HAZ. Contrast p values were adjusted for multiple comparisons using the Tukey method. Mixed effect models were used to assess changes in *z*-scores associated with time, daily steroid exposure, and age at diagnosis. This analysis evaluated the 48-month mark as data were available for 41/61 (67%) patients, while we only had 26/61 (43%) patients at 60 months. Most of the 32 participants missing the 60-month visit were patients who reached 18 years of age (19/32, 59%) and were transferred to adult care. Patients with missing visits are tallied in Supplemental Table S1, http://links.lww.com/HC9/B875. Kaplan-Meier survival methods were used to estimate the cumulative incidence of new-onset growth impairment. These consider only the first onset of growth impairment and do not reflect persistence or resolution. The log-rank test was used to assess the relationship with covariates (gestational age, AIH type, IBD, social worker involvement, estimated pubertal status, sex, >10 mg daily prednisone 6 months after treatment initiation, ALT normalized after 6 months, and overweight status at diagnosis). A time-dependent Cox proportional hazards model was constructed to determine predictors for growth impairment. The same covariates were evaluated, including age at diagnosis. Covariates were retained in the multivariate analysis if they were significant in univariate analysis or per clinical judgment (ie, age and sex). Spearman rank correlation tested the relation between age and histological scores at diagnosis. *p* value of <0.05 was considered statistically significant. Data analysis and visualizations were performed using IBM SPSS statistics 29.0.1.0 and R (version 4.3.1).

## RESULTS

**FIGURE 1:**
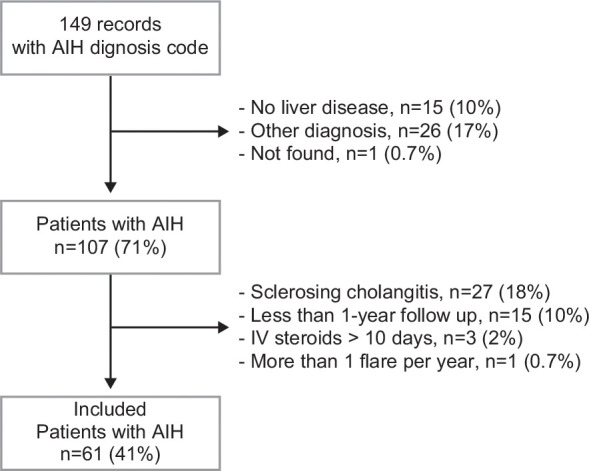
Flowchart for patients’ selection. Patients carrying the code of “autoimmune hepatitis” at The Hospital for Sick Children between January 1, 1997 and January 31, 2023. Abbreviation: AIH, autoimmune hepatitis.

One hundred and forty-nine patients were identified from the SickKids electronic medical charting system between January 1, 1997, and January 31, 2023. Twenty-seven patients (18%) were excluded due to sclerosing cholangitis, and 15 (10%) were excluded due to short follow-up (<1 year). Patient selection is described with a flow chart in Figure 1. Overall, 61 patients were included in the analysis, and their characteristics are presented in Table 1. There was a female predominance (n = 40, 66%), and the median age at diagnosis was 11.5 years (IQR: 8.4, 13.8). Most patients had AIH type 1 (n = 53, 87%) and with a median age of 11.8 (IQR: 9.6–14.3), these children were older than patients diagnosed with AIH type 2 (median age 6.3, IQR: 2.9–12.9, *p* = 0.029). The median age at the last follow-up was 15.9. Median ALT at diagnosis was 1025 units/L (IQR: 532–1601), and after 6 months of therapy, it dropped to 50 (IQR: 25–91), as expected (Supplemental Figure S1, http://links.lww.com/HC9/B875). Only 1 patient was documented to be taking amlodipine, among the other medications reviewed. As for second-line agents, 7 patients were switched to MMF (11.4%). One of them was switched to tacrolimus as a third agent for poor response and failure to achieve biochemical remission.

**TABLE 1:** Baseline characteristics of children diagnosed with autoimmune hepatitis treated at The Hospital for Sick Children

Demographics	All (n = 61)	Disease characteristics at diagnosis	All (n = 61)
Biological sex, female, n (%)	40 (66)	AIH type, n (%)
Gestation age at birth, n (%)		AIH type 1	53 (87)
Term	31 (51)	AIH type 2	8 (13)
Preterm	2 (3)	ALF presentation, n (%)	1 (2)
Unknown	28 (46)	AST (U/L)	1107 (601–1861)
Ethnicity, n (%)		ALT (U/L)	1025 (532–1601)
North American/ European	18 (30)	IgG (g/L)	26 (19–38)
Latin	3 (5)	GGT (U/L)	94 (70–147)
African	8 (13)	Conjugated bilirubin (μmol/L)	6 (0–73)
Asian	13 (21)	Total bilirubin (μmol/L)	56 (12–144)
Unknown	21 (34)	Albumin (g/L)	42 (37–44)
Age at diagnosis, y	11.5 (8.4, 13.8)	INR	1.2 (1.15–1.4)
IBD, n (%)	3 (5)	Average prednisone exposure	0.56 (0.43–0.68)
Social worker involved, n (%)	12 (20)	over 6 mo (mg/kg/d)	

*Note:* Data presented as n (%) or median and interquartile range.

Abbreviations: AIH, autoimmune hepatitis; ALF, acute liver failure; IBD, inflammatory bowel disease; INR, international normalized ratio.

In our cohort, 20 patients were still treated with >10 mg daily prednisone after 6 months. The median dose of steroids after 6 months for these patients was 15 mg (IQR: 15–20, range: 12.5–20 mg daily). There was no difference between this group and the group treated with <10 mg at 6 months, in terms of biological sex, gestational age at birth, social worker involvement, IBD overlap, type of AIH, number of flares, activity grade, and fibrosis stage at the time of the diagnosis as seen in the first liver biopsy. These patients were, however, older at diagnosis (12.2, IQR: 10.8–15.3, vs. 10.5, IQR: 7.2–13.5, *p* = 0.04) and had higher weight (45.8, IQR: 39.2–55.6, vs. 35.3 kg, IQR: 24.5–53.2, *p* = 0.03,); thus, their initial steroid dose was higher.

### Ponderal gains and slowed linear growth do not return to baseline 4 years after treatment

**FIGURE 2:**
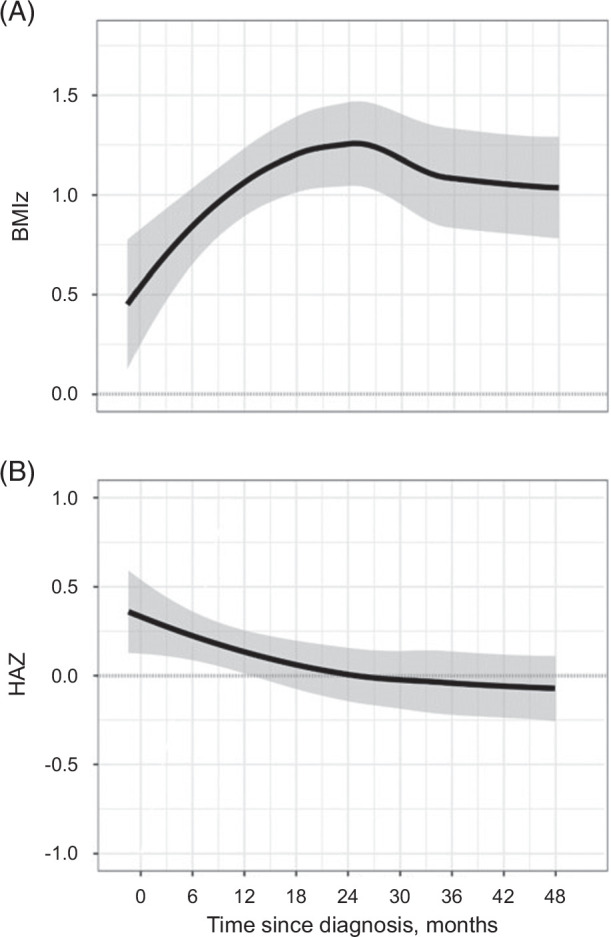
Growth patterns in children with autoimmune hepatitis treated with standard-of-care therapy. Overall trajectory in children with AIH (n = 61) for BMIz (A, top panel) and height-for-age *z*-scores (B, bottom panel). Group trajectories fitted by locally estimated scatterplot smoothing (LOESS), gray shadow indicates CIs. Abbreviations: AIH, autoimmune hepatitis; BMIz, body mass index *z*-score; HAZ, height-for-age *z*-score.

Based on model fit criteria, a knot point indicating a change in slope was positioned at 6 months for BMIz trajectory models and at 12 months for HAZ. We tested whether growth in children receiving standard of care for AIH differed between periods before or after these knot points (Figure 2, Table 2 and, full models presented in Supplemental Tables S2 and S3, http://links.lww.com/HC9/B875). BMIz showed a sharp monthly increase in the first 6 months (slope: 0.19 BMIz/mo [95% CI: 0.15, 0.22], *p* < 0.0001), during which BMIz increased overall by 1.1 *z*-score (95% CI: 0.85, 1.4). After 6 months, the gain in BMIz showed a small monthly decrease (slope after 6 mo: −0.011 BMIz/month [95% CI: −0.017, −0.0056], *p* = 0.00011). By 4 years after treatment initiation, the BMIz of children receiving SOC treatment was higher than baseline by 0.64 BMIz (0.19, 1.1), *p* = 0.0019. HAZ declined significantly in the first 12 months (slope: −0.023 HAZ/mo [95% CI: −0.033, −0.013], *p* < 0.0001) and showed no significant “catch-up” or compensatory increase thereafter; the slope after 12 months did not differ from 0 (slope: −0.0020 HAZ [−0.0061, 0.0022], *p* = 0.36). Four years after treatment initiation, the HAZ of children was lower by 0.35 *z*-score (95% CI: 0.12, 0.57), *p* < 0.001. Each 1 mg/kg/d increase in prednisone exposure increased BMIz gain in the first 6 months by 0.27 (95% CI: 0.11, 0.42), *p* = 0.001, whereas for each 1 mg/kg/d increase of exposure HAZ tended to decline more by −0.02 over the first 6 months (95% CI: −0.03, −0.01), *p* = 0.0054. These effects were more pronounced in patients treated with a higher dose of Prednisone (Supplemental Figure S2, http://links.lww.com/HC9/B875). Results were near-identical with adjustment for age and age of diagnosis (Supplemental Tables S2 and S3, http://links.lww.com/HC9/B875).

**TABLE 2:** Monthly changes in body mass index and height for age *z*-scores during the early and late periods posttreatment initiation in children with autoimmune hepatitis treated with standard SOC treatment

BMIz						
Months	Est.	95% CI	Slope	Est.	95% CI	*p* value
0	0.21	[−0.07, 0.49]	Slope before 6 mo.	0.19	[0.15, 0.22]	<0.0001
6	1.33	[−1.0, 1.7]	Slope after 6 mo.	−0.011	[−0.017, −0.0056]	0.00011
12	1.26	[0.93, 1.6]				
48	0.85	[0.42, 1.3]	Contrast between months	Est.	95% CI	*p* value
			0 vs. 6	−1.1	[−1.4, −0.85]	<0.0001
			0 vs. 48	−0.64	[−1.1, −0.19]	0.0019
HAZ						
Months	Est.	95% CI	Slope	Est.	95% CI	*p* value
0	0.34	[−0.073, 0.61]	Slope before 12 mo.	−0.023	[0.15, 0.22]	<0.0001
6	0.20	[−0.057, 0.46]	Slope after 12 mo.	−0.0020	[−0.0061, −0.0022]	0.36
12	0.065	[−0.20, 0.33]				
48	−0.0052	[−0.27, 0.26]	Contrast between months	Est.	95% CI	*p* value
			0 vs. 12	0.28	[0.11, 0.45]	0.00032
			0 vs. 48	0.35	[0.12, 0.57]	0.00080

*Note*: Anthropometry trajectories were modeled using piecewise linear mixed models. Model-derived marginal means were calculated at 0, 6, 12, and 48 months. Monthly changes in BMIz (slopes) were estimated for the early and late periods after treatment initiation (ie, before and after 6 mo). Monthly changes in HAZ (slopes) were estimated before and after 12 months. Knot positions for BMIZ and HAZ were chosen based on model fit metrics. Contrasts between time points were evaluated using emmeans R package, and *p* values were adjusted for multiple comparisons using the Tukey method. Full model results are presented in Supplemental Tables S2 and S3, http://links.lww.com/HC9/B875.

Abbreviations: BMIz, body mass index *z*-score; HAZ, height-for-age *z*-score; SOC, standard-of-care.

### Growth impairments

**FIGURE 3:**
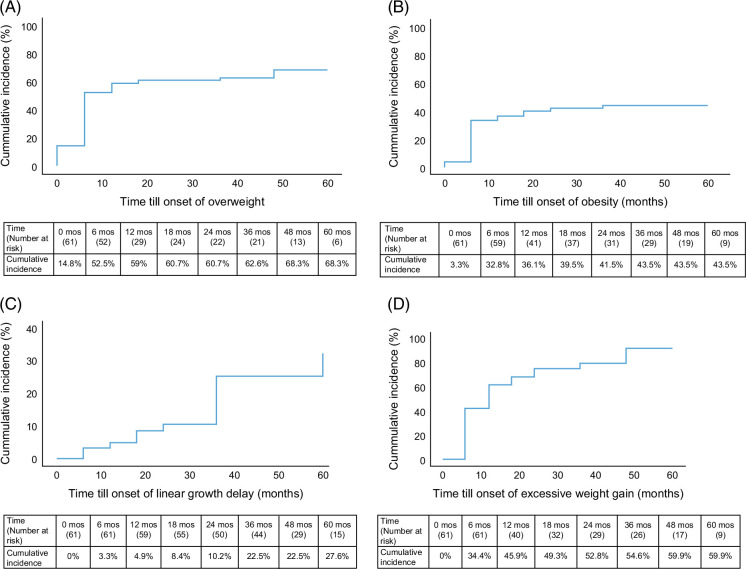
Cumulative incidence of growth impairments 5 years after the diagnosis: (A) overweight status (BMIz >1); (B) obesity (BMIz >1.64); (C) linear growth delay (HAZ reduced at >1 SD); (D) excessive weight gain (BMIz increased at >1 SD). Abbreviations: BMIz, body mass index for age and sex *z*-score; HAZ, height for age and sex *z*-score.

The point prevalence of overweight status (BMIz >85% or >1.64) at diagnosis and after 5 years was 18% (n = 11/61) and 42% (n = 11/26), respectively. At diagnosis, 4.9% (n = 3/61) were obese, and 27% (n = 7/26) were obese after 5 years. The estimated cumulative incidence of overweight status was 39% at 1 year and 68% at 5 years (Figure 3A). The estimated cumulative incidence of obesity was 36% at 1 year and 43% at 5 years (Figure 3B). There was no difference in the prevalence of overweight and obesity between males and females (*p* = 0.2, *p* = 0.4).

**FIGURE 4:**
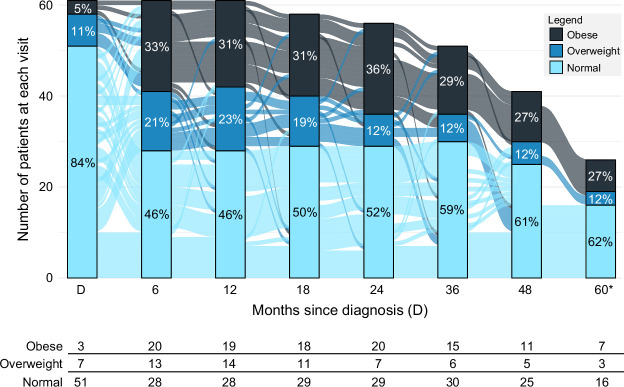
The alluvial plot shows group mobility between “normal,” “obese,” and “overweight” categorization over time based on the BMI-for-age *z*-score in children with autoimmune hepatitis receiving standard-of-care therapy. The *x*-axis represents months since diagnosis (D), and the *y*-axis represents the count of patients in each category colored as per the legend. Each flow line indicates a child's trajectory and how they change categorization between time points. Percentages detail the group categorization at each time point.

Thirteen children had new-onset linear growth delay (21.3%, Figure 3C). The estimated cumulative incidence of linear growth delay within 1 year of diagnosis was 4.9%. It was 22.5% within 3 years, and 27.6% within 5 years. Thirty-five (57.4%) patients developed excessive weight gain in 5 years. The estimated cumulative incidence was 46% within 1 year and 60% within 5 years (Figure 3D). Obesity and overweight prevalence over time are presented with alluvials in Figure 4. Also, the weight trajectory across visits for each patient, including missing data, is presented in Supplemental Figure S3, http://links.lww.com/HC9/B875.

**FIGURE 5:**
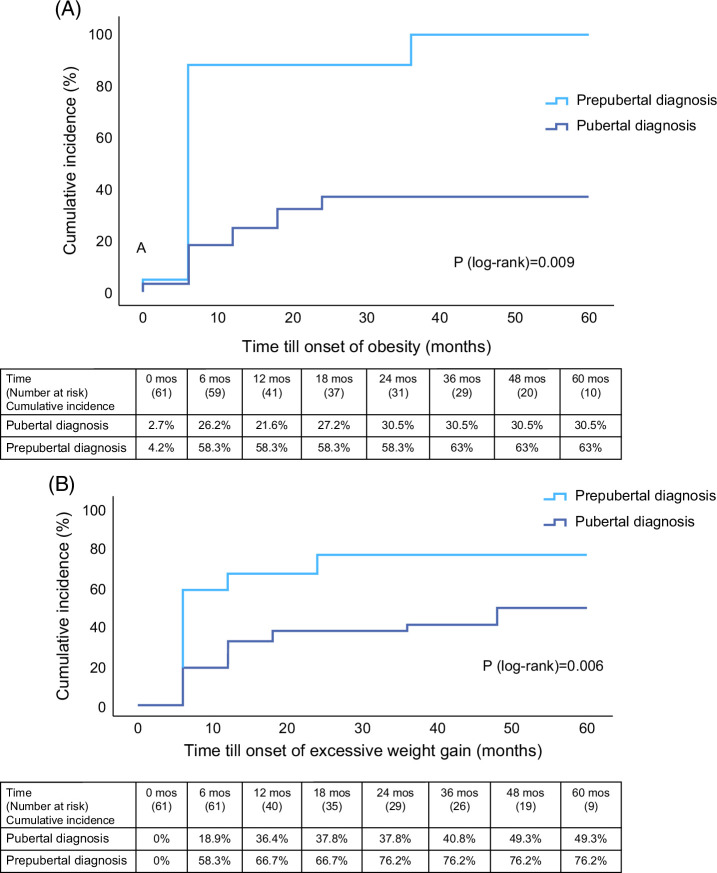
Time till onset of obesity (A) and excessive weight gain (B) in patients diagnosed before puberty onset and after. Obesity, BMI *z*-score >1.64; excessive weight gain, BMI *z*-score increase >1. Abbreviation: BMI, body mass index.

The estimated cumulative incidence of excessive weight gain or obesity at 5 years was 76% and 63% among patients diagnosed prepubertally, compared to 49% and 30% among patients diagnosed after the onset of puberty (log-rank test *p* < 0.01 and *p* < 0.01 respectively), as seen in Figure 5.

### Predictors for growth impairments

Table 3 presents factors associated with growth impairments after Cox regression analysis. In the multivariate analysis, linear growth delay was associated with young age at diagnosis and prednisone daily dose of >10 mg 6 months after initiation of treatment. The risk of developing linear growth delay decreased by HR 0.7 ([95% CI: 0.5–0.9], *p* = 0.002) for every 1-year increase in age at the time of diagnosis. Importantly, there was no correlation between age and activity score (*r* = 0.22, *p* = 0.09), and age and fibrosis stage (*r* = 0.23, *p* = 0.11) at the time of the diagnosis.

**TABLE 3:** Variables associated with growth impairments: Multivariate analysis

	Linear growth delay, HR				Excessive weight gain, HR				Overweight, HR				Obesity, HR			
	Unadjusted		Adj.		Unadj.		Adj.		Unadj.		Adj.		Unadj.		Adj.	
Variable	(95% CI)	*p*	(95% CI)	*p*	(95% CI)	*p*	(95% CI)	*p*	(95% CI)	*p*	(95% CI)	*p*	(95% CI)	*p*	(​​​​​​95% CI)	*p*
Age at diagnosis	0.8 (0.7–0.9)	0.005	0.7 (0.5–0.8)	<0.001	0.9 (0.8–1.0)	0.06	0.9 (0.8–1)	0.1	1 (0.9–1.1)	0.85	1 (0.9–1.1)	0.9	0.9 (0.8–1.0)	0.059	0.9 (0.8–1)	
Female	0.9 (0.3–2.7)	0.82	0.4 (0.1–1.4)	0.1	1.6 (0.7–3.3)	0.25	1.4 (0.7–3.1)	0.4	1.1 (0.5–2.0)	0.87	1 (0.5–2.1)	0.9	1 (0.4–2.2)	0.95	0.8 (0.4–1.8)	
Daily prednisone >10 mg 6 mo after treatment was initiated	2.6 (0.9–7.8)	0.085	10.5 (2.4–45.8)	0.002	0.8 (0.4–1.6)	0.54	0.9 (0.4–2)	0.9	0.9 (0.5–1.8)	0.87	1 (0.5–1.9)	0.9	0.7 (0.3–1.6)	0.38	1 (0.4–2.6)	
Overweight at diagnosis													4.7 (2.1–10.8)	<0.001	4.7 (2–10.9)	

*Note*: Excessive weight gain, BMI *z*-score increased >1 *z-*score; linear growth delay, height *z*-score reduced >1 SD; obesity, BMI *z*-score >1.64; overweight, BMI *z*-score >1.

Abbreviation: BMI, body mass index.

For patients still receiving more than 10 mg prednisone daily 6 months after treatment was initiated, the risk of developing linear growth delay was associated with an HR of 10.2 (95% CI: 2.1–50.1). Patients who met the criterion for overweight status at diagnosis had a 5.2-fold (95% CI: 2.1–12.6) increased risk of developing obesity. Prematurity, IBD overlap, social worker involvement, AIH type, and number-of-flares were not associated with growth impairments.

## DISCUSSION

In this study, we explored the long-term impacts of SOC therapy on growth in children with AIH. Our findings indicate that these children experience long-term excessive weight gain, obesity, and linear growth delay. Specifically, their growth trajectories were altered, with higher BMI and lower HAZ observed 4 years post-therapy initiation compared to baseline. Furthermore, younger age at diagnosis and a daily dose of more than 10 mg, 6 months after the diagnosis was associated with greater linear growth delay. Children who were overweight at diagnosis were more likely to develop obesity. In addition, being diagnosed before the onset of puberty increases the risk of excessive weight gain and obesity. These findings underscore the importance of closely monitoring growth parameters in children undergoing SOC therapy for AIH, and they highlight the need for alternative treatment options to mitigate these long-term effects.

These results provide valuable insights into how SOC therapy impacts longer-term BMI trajectories of pediatric patients with AIH. The strong increase in BMI*z* during the initial 6 months posttreatment initiation is not followed by a sufficient decrease in BMI*z* to allow a return to baseline. This underscores a pattern of sustained long-term weight gain. We then focused on the children who experienced excessive weight gain (gained more than 1 BMI*z*), overweight (BMI*z* >1), and obesity (BMI*z* >1.64). The cumulative incidence of obesity increased by 40% in the 5 years since treatment was initiated, with more than half of the children classified as overweight. These findings highlight a concerning trend of increasing prevalence of excessive weight gain over time. A study performed based on medical records of Ontarian children for the years 2014–2015 found that 16.3% of children were overweight and 8.6% were obese.^1^ In comparison, the children from our study have overall 16% overweight/obesity rates, but after 4–5 years of treatment, their overweight/obesity rates climb to higher levels (39%) compared to the highest estimate.

This pattern of impaired growth associated with steroid induction has been observed in other conditions such as juvenile idiopathic arthritis,^12^ and nephrotic syndrome,^15^,^16^ but in this series we show for the first time the irreversible effect of steroid induction on weight gain patterns in patients with AIH. This broader context underscores the importance of monitoring and addressing potential growth impairments in pediatric patients undergoing steroid induction for various medical conditions, including AIH. Practically, when this pattern is recognized, we recommend targeted education for the patient and family regarding necessary lifestyle modifications and referral to a dietician.

Analysis of specific covariates and their association with overweight status, obesity, and excessive weight gain in children with AIH provides important insights for clinical practice. First, it is notable that factors such as age at diagnosis, gestational age at birth, AIH type, presence of IBD, social worker involvement, estimated pubertal status, sex, and ALT normalization after 6 months were not associated with overweight status, obesity, and excessive weight gain. The only variable found to predict obesity was overweight status at diagnosis. This finding emphasizes the importance of identifying at-risk patients and that more aggressive steroid weaning protocols should be considered in patients who are already overweight at diagnosis. In addition, the association between diagnosis before the onset of puberty and increased risk for excessive weight gain and obesity is consistent with findings from studies on steroid use in the treatment of acute lymphoblastic leukemia.^16^,^17^,^18^,^19^ This highlights the need for proactive measures to manage weight gain in pre-pubescent patients diagnosed with AIH.

This study also investigated the effect of SOC therapy on linear growth. Growth trajectories for height declined in the first 12 months after treatment initiation, and no subsequent catch-up growth was observed. Although others have described the decline in HAZ with minimal recovery in AIH,^6^ our data identify and highlight the magnitude and the prevalence of patients exhibiting linear growth delay (>1 decrease SD in HAZ) following steroid induction for AIH. We found that younger patients at diagnosis carried a greater risk for linear growth delay, which has been previously described in other diseases.^13^,^20^,^21^ Interestingly, correlation between age and initial activity score (*r* = 0.22, *p* = 0.09), and between age and fibrosis stage (*r* = 0.23, *p* = 0.11), was very weak and not significant. Hence, we do not believe the reduced linear growth in the younger age group is explained by more aggressive disease in this age group. In addition, children were not stunted at diagnosis when disease activity would be highest. HAZ for the younger children (0.2, IQR: −0.4, −1.3) was not lower than the older children (0.2, IQR: −0.4, 0.75, *p* = 0.76), and there was no correlation between age at diagnosis and HAZ at diagnosis (*r* = −0.39, *p* = 0.61).

In our analysis, a daily prednisone dose of more than 10 mg, at 6 months after diagnosis also predicted linear growth delay. This was consistent with another retrospective study of 37 children with AIH, which showed that a higher cumulative dose of steroids was associated with lower HAZ.^7^ These findings can be explained by the pathomechanism underlying linear growth impairment with steroid use.^21^,^22^ Steroids reduce growth by altering bone remodeling in a manner that favors bone resorption through various pathways, including suppression of growth hormone through stimulation of somatostatin, inhibition of gonadotrophic and sex hormones, and induction of secondary hyperparathyroidism through increased calcium loss in the kidney and in the gut. Loss of bone and deterioration in short-term growth are dependent on the dose of corticosteroids and occur most prominently over the first 6–12 months of therapy,^22^,^23^,^24^,^25^,^26^ as also seen in our cohort. Growth reduction seems to be maximal during the first year of therapy and less pronounced in subsequent years of treatment.

According to AASLD guidelines, patients dose is weaned to 10 mg daily over the course of the first 6 months of treatment.^3^ One-third of patients in this cohort were receiving higher doses at this time point. These patients did not differ in their biological sex, gestational age at birth, social worker involvement, overlap with IBD, type of AIH, frequency of flares, activity grade, and fibrosis stage at the time of diagnosis, but they were older and their weight was higher. We propose that this explains the extended weaning period observed in this group. We acknowledge the discrepancy between guideline recommendations to taper prednisone to 5–10 mg daily by 6 months and clinical practice as described in this paper. We strongly advocate for close follow-up during the first 6 months of treatment to facilitate more rapid steroid tapering. However, it is important to recognize that in older and heavier patients starting with high-dose prednisone (eg, 60 mg/d), the tapering process can be prolonged, even when the biochemical response is achieved. At our center and as per the guidelines, we generally aim to start the weaning after 4–8 weeks,^1^ and reduce the dose from 60 to 40 mg/d in 10 mg increments every 2 weeks, then from 40 to 20 mg/d in 5 mg increments every 2 weeks, and finally taper from 20 in 2.5–5 mg decrements every 2–4 weeks (overall 18–34 wk). In many cases, this weaning period naturally extends beyond the expected 26-week timeline due to factors such as fluctuations in blood work, illness, holidays, or travel. Notably, this extended steroid tapering, even in patients who achieve complete biochemical remission, underscores our argument regarding the long-term use of steroids in SOC therapy. The extended duration of treatment may contribute to the development of obesity and growth delay as described, further supporting our concerns about the metabolic consequences of long-term steroid therapy in this population.

Chronic inflammation is another potential contributor to the linear growth delay. Long-term elevated circulating levels of cytokines might cause a reduction in IGF-1, and as a result, linear growth will be delayed.^27^ In our cohort, ALT normalization after 6 months was not associated with linear growth delay. ALT normalization is one of the markers for remission in patients with AIH; hence, we feel chronic inflammation is less likely to play a role here.

Our study has several strengths and limitations. It is a retrospective study, conducted in a single but large tertiary center with hepatic disease specialization. Despite being the largest liver clinic in Canada, the sample size achieved was still relatively small due to the rarity of AIH. Another limitation is that patients were not followed into adulthood and data were unavailable once they transitioned to adult care facilities at 18 years of age. Some patients with chronic inflammatory disease can continue to grow beyond the age of 18 years.^28^ Also, other side effects of corticosteroid treatment were not assessed in this study, including glucose intolerance, bone health issues like osteopenia, and increased prevalence of fractures, which were observed in retrospective studies for other diseases.^29^,^30^,^31^,^32^ Parameters such as fasting glucose, HbA1C, bone density scan, and parental auxology would be indeed beneficial but were not available in the patient charts. Finally, we recorded the steroid exposure in the first 6 months of therapy and recorded the number of flares after diagnosis, but we did not have details of the steroid weaning process for each patient who continued to receive steroid treatment after 6 months.

## CONCLUSIONS

Our findings indicate that children diagnosed with AIH receiving SOC therapy with prednisone induction demonstrate long-term excess weight gain, obesity, and linear growth delay. Both BMI and height trajectories were impaired, even 4 years after treatment initiation. Young age at diagnosis and >10 mg of daily prednisone at 6 months are predictors for linear growth delay. Patients who are overweight at diagnosis are more likely to become obese. Patients diagnosed before puberty are more prone to excessive weight gain and obesity. These findings provide cautionary evidence for clinicians managing pediatric patients with AIH, emphasizing the importance of individualized monitoring to address weight-related issues during treatment. These data highlight the need for clinical teams to serially re-evaluate standard treatment algorithms for pediatric AIH in terms of steroid dosing and potential nonsteroid alternatives for induction therapy.

## Supplementary Material

SUPPLEMENTARY MATERIAL
