# Supplementary material for: Children with autoimmune hepatitis receiving standard-of-care therapy demonstrate long-term obesity and linear growth delay
Source: Hepatol Commun. 2025 Feb 3;9(2):e0624. doi: 10.1097/HC9.0000000000000624 (PMC12333776; doi:10.1097/HC9.0000000000000624)
Supplement: SUPPLEMENTARY MATERIAL [file hc9-9-e0624-s001.pdf]

Supplemental Table 1: Tally of children that did not complete the 8 visits.

#	Completed visits	Follow up time (months)	Age at last visit	Reason
1	3	13.3	17.8	G
2	3	10.2	18.0	G
3	4	19.7	17.8	G
4	5	29.7	17.3	G
5	5	26.3	17.8	G
6	5	25.0	17.9	G
7	5	36.5	19.9	G
8	6	35.2	17.2	G
9	6	32.2	17.5	G
10	6	35.3	17.9	G
11	6	42.1	18.0	G
12	6	30.4	18.2	G
13	6	36.6	18.8	G
14	7	45.3	17.2	G
15	7	53.8	17.3	G
16	7	50.4	17.3	G
17	7	48.8	17.6	G
18	7	50.6	18.0	G
19	7	42.6	18.1	G
1	3	14.7	9.6	Missed
2	5	32.6	12.6	Missed
3	6	52.1	9.2	Missed
4	6	44.1	9.6	Missed
5	6	36.5	11.8	Missed
6	6	38.6	13.7	Missed
7	6	35.3	16.1	Missed

8	7	53.8	10.2	Missed
9	7	57.1	12.8	Missed
10	7	47.0	13.8	Missed
11	7	57.8	15.3	Missed
12	7	52.7	15.4	Missed
13	7	51.4	15.8	Missed

The table presents the number of visits, follow-up times and age at last clinic visit for every patient individually. The upper part is for patients who graduated and transitioned to the adults' care, and the lower part is for patients who stopped follow up in SickKids prior to transition. G - Graduation.

Supplemental Table 2. Body mass index z-scores modelled using piecewise mixed effect regression.

**BMIz**	**Base**			**Prednisone**			**Prednisone with adjustment**		
Predictors	*Est.*	*95% CI*	*p*	*Est.*	*95% CI*	*p*	*Est.*	*95% CI*	*p*
(Intercept)	0.21	-0.06, 0.48	0.128	1.1	0.29 – 1.92	**0.008**	2.19	0.70 – 3.68	**0.004**
Slope between 0- 6 month	0.19	0.15, 0.22	**<0.001**	0.03	-0.06 – 0.12	0.525	0.03	-0.06 – 0.12	0.531
Slope change before and after 6 months	-0.2	-0.23, -0.17	**<0.001**	-0.04	-0.13 – 0.06	0.442	-0.04	-0.13 – 0.06	0.448
Average prednisone exposure (main effect)				-1.53	-2.84 – -0.22	**0.023**	-2.01	-3.46 – -0.56	**0.007**
Slope change before 6 months × Average prednisone exposure				0.27	0.11 – 0.42	**0.001**	0.27	0.12 – 0.42	**0.001**
Slope change after 6 months × Average prednisone exposure				-0.28	-0.43 – -0.12	**<0.001**	-0.28	-0.43 – -0.12	**<0.001**
Age at diagnosis							-0.06	-0.14 – 0.01	0.112
Sex							-0.2	-0.74 – 0.34	0.457
**Random Effects**									
σ^2	0.16			0.16			0.16		
τ_{00}	1.02 study_id			0.93 study_id			0.88 study_id		
τ_{11}	0.01 id.before 6 mo.			0.01 id.before 6 mo.			0.01 id.before 6 mo.		
	0.01 id.after 6 mo.			0.01 id.after 6 mo.			0.01 id.after 6 mo.		
ρ_{01}	0.07			0.26			0.2		
	-0.05			-0.26			-0.21		
ICC	0.92			0.92			0.92		
Observations	411			411			411		
Marginal R^2 / Conditional R^2	0.063 / 0.927			0.069 / 0.927			0.096 / 0.923		

Table presents results for both fixed and random terms of mixed effect piecewise models fit using *lme4* R package. Different random structures were tested and including terms for random slope per participant improved model fit. A knot point was either omitted or included (positioned at either 6- or 12 months) to define distinct phases of growth. Based on fit criteria the knot point for BMIz was positioned at 6 months post-treatment initiation. Time was coded in months. Final models were fit with restricted maximum likelihood. BMIz, body mass index z-score.

Supplemental Table 3. Height for age z-scores modelled using piecewise mixed effect regression.

**HAZ**	**Base**			**Prednisone**			**Prednisone with adjustment**		
Predictors	*Est.*	*95% CI*	*p*	*Est.*	*95% CI*	*p*	*Est.*	*95% CI*	*p*
(Intercept)	0.34	0.08, 0.61	**0.011**	0.83	0.02, 1.63	**0.044**	1.12	-0.29, 2.52	0.12
Slope 0-12 months	-0.02	-0.03, -0.01	**<0.001**	0.01	-0.02, 0.04	0.677	0.01	-0.02, 0.04	0.674
Slope change before and after 12 months	0.02	0.01, 0.03	**0.001**	-0.02	-0.06, 0.02	0.291	-0.02	-0.06, 0.02	0.289
Average prednisone exposure (main effect)				-0.83	-2.13, 0.47	0.212	-1.18	-2.62, 0.25	0.106
Slope change before 12 months × Average prednisone exposure				-0.05	-0.10, -0.00	**0.05**	-0.05	-0.10, -0.00	**0.05**
Slope change after 12 months × Average prednisone exposure				0.07	0.01, 0.13	**0.021**	0.07	0.01, 0.13	**0.021**
Age at diagnosis							-0.03	-0.10, 0.04	0.434
Sex							0.34	-0.16, 0.84	0.183
**Random Effects**									
σ^2	0.04			0.04			0.04		
τ_{00}	1.06 $_{id}$			1.03 $_{id}$			1.01 $_{id}$		
τ_{11}	0.00 $_{id.before\ 12\ mo.}$			0.00 $_{id.before\ 12\ mo.}$			0.00 $_{id.before\ 12\ mo.}$		
	0.00 $_{id.after\ 12\ mo.}$			0.00 $_{id.after\ 12\ mo.}$			0.00 $_{id.after\ 12\ mo.}$		
ρ_{01}	-0.21			-0.27			-0.24		
	0.1			0.17			0.12		
ICC	0.96			0.96			0.96		
Observations	411			411			411		
Marginal R^2 / Conditional R^2	0.014 / 0.962			0.062 / 0.962			0.093 / 0.963		

Table presents results for both fixed and random terms of mixed effect piecewise models fit using *lme4* R package. Different random structures were tested and including terms for random slope per participant improved model fit. A knot point was either omitted or included (positioned at either 6- or 12 months) to define distinct phases of growth. Based on fit criteria the knot point for BMIz was positioned at 12 months post-treatment initiation. Time was coded in months. Final models were fit with restricted maximum likelihood. HAZ, height-for-age z-score.

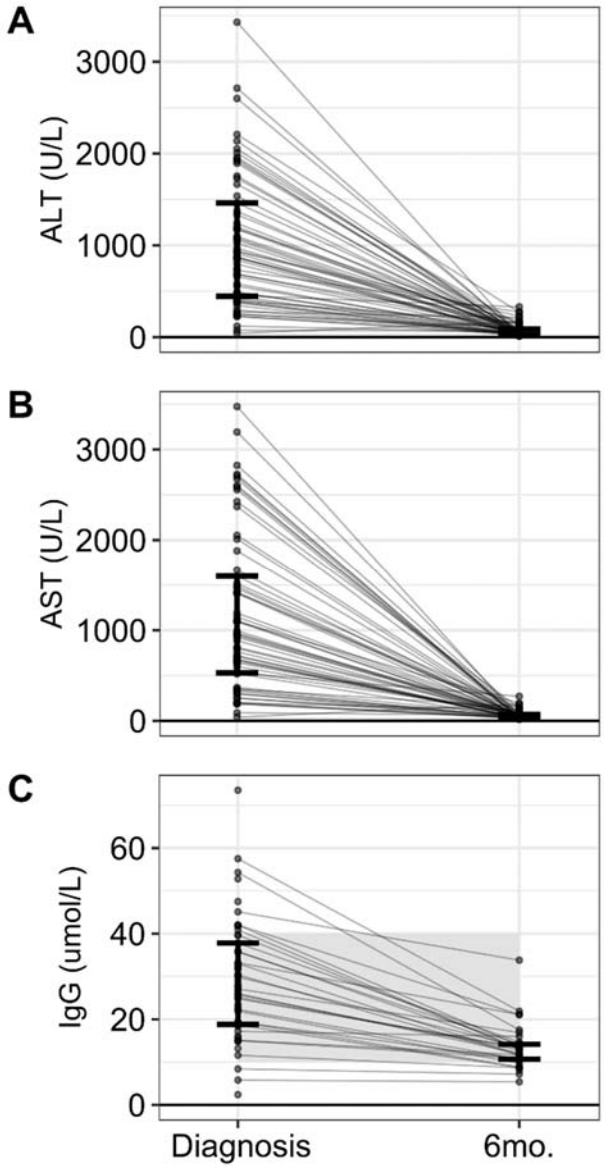

Supplemental Figure 1. Serum liver biochemistry in children with AIH at diagnosis and after 6 months of treatment initiation. A) ALT B) AST and C) IgG. Cross bars indicate interquartile range. Grey shadow box indicates normal range of marker. ALT – Alanine transaminase. AST – aspartate aminotransferase. IgG – Immunoglobulin G.

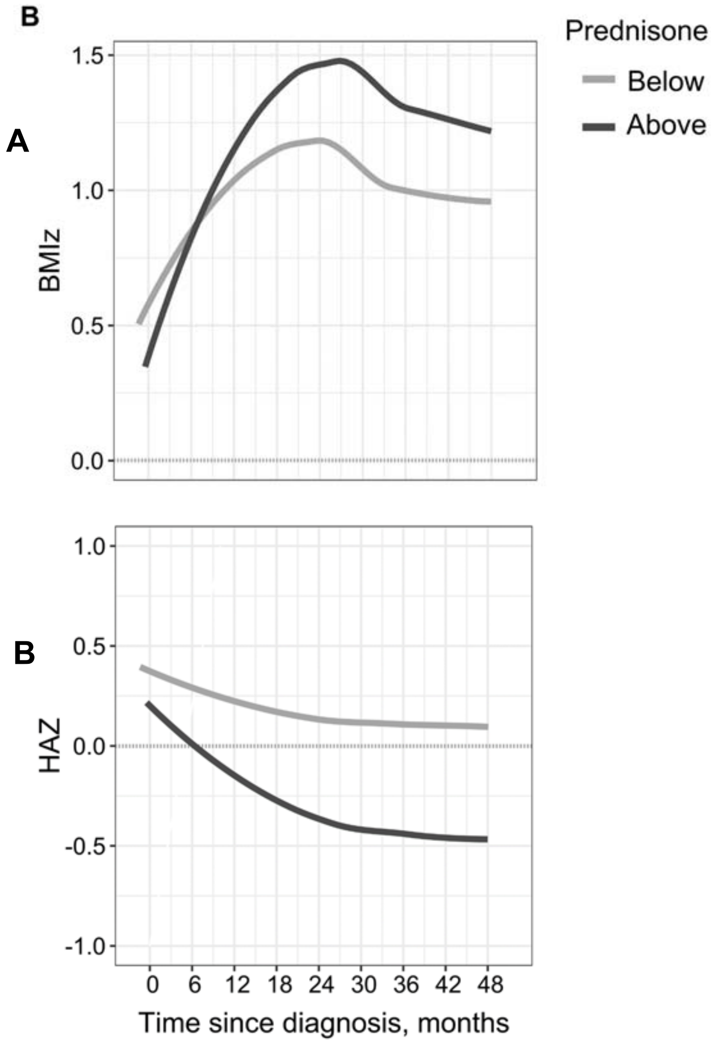

Supplemental Figure 2. Growth patterns in children with autoimmune hepatitis treated with standard-of-care therapy divided by steroid exposure. Trajectories split by children who were exposed to top quartile of prednisone, i.e., exposure ≥ 0.69 units, (n=15,

dark grey) versus those below (n=46, light grey) for BMIz (B, top panel) and height-for-age (B, bottom panel). Group trajectories fitted by locally estimated scatterplot smoothing (LOESS). BMIz, body mass index z-score; HAZ, height-for-age z-score.

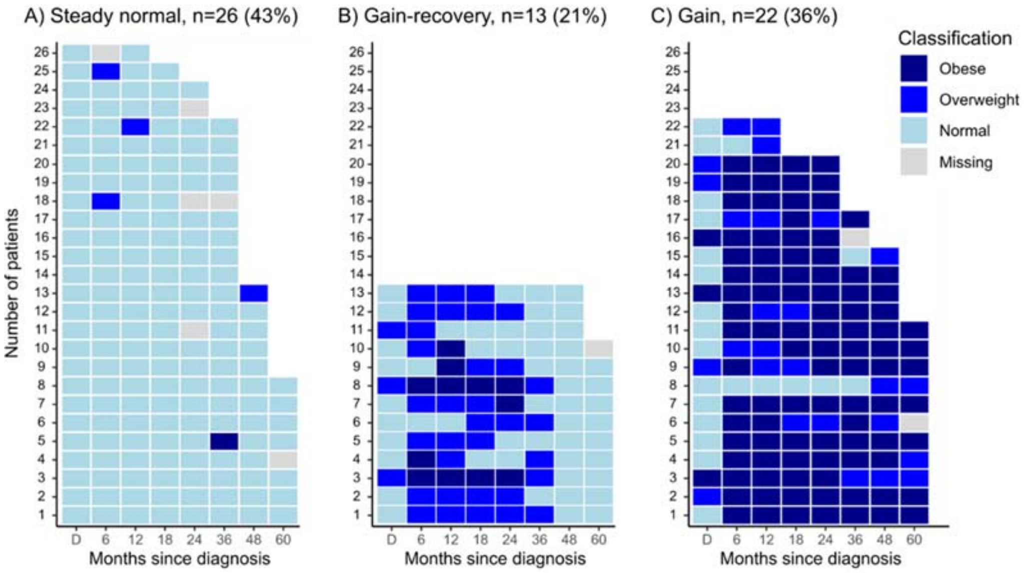

Supplemental Figure 3. Weight trajectory across visits for each patient that A) showed a general steady normal weight pattern; B) a pattern of gain followed by indication of recovery; and C) a pattern of gain by the end of follow up. Adiposity classifications are as indicated by legend, and missing BMI data (n=9) are in grey.
